# The 11S globulin Sin a 2 from yellow mustard seeds shows IgE cross-reactivity with homologous counterparts from tree nuts and peanut

**DOI:** 10.1186/2045-7022-2-23

**Published:** 2012-12-11

**Authors:** Sofía Sirvent, Martial Akotenou, Javier Cuesta-Herranz, Andrea Vereda, Rosalía Rodríguez, Mayte Villalba, Oscar Palomares

**Affiliations:** 1 Department of Biochemistry and Molecular Biology, School of Chemistry, Complutense University of Madrid, Madrid, 28040, Spain; 2 Service of Allergy, IIS-Fundación Jiménez Díaz, Madrid, Spain

**Keywords:** Food allergy, Mustard allergy, Tree nut allergy, Peanut allergy, Cross-reactivity, 11S globulins, Sin a 2, lgG/IgE epitopes

## Abstract

**Background:**

The 11S globulin Sin a 2 is a marker to predict severity of symptoms in mustard allergic patients. The potential implication of Sin a 2 in cross-reactivity with tree nuts and peanut has not been investigated so far. In this work, we studied at the IgG and IgE level the involvement of the 11S globulin Sin a 2 in cross-reactivity among mustard, tree nuts and peanut.

**Methods:**

Eleven well-characterized mustard-allergic patients sensitized to Sin a 2 were included in the study. A specific anti-Sin a 2 serum was obtained in rabbit. Skin prick tests (SPT), enzyme-linked immunosorbent assay (ELISA), immunoblotting and IgG or IgE-inhibition immunoblotting experiments using purified Sin a 2, Sin a 1, Sin a 3, mustard, almond, hazelnut, pistachio, walnut or peanut extracts were performed.

**Results:**

The rabbit anti-Sin a 2 serum showed high affinity and specificity to Sin a 2, which allowed us to demonstrate that Sin a 2 shares IgG epitopes with allergenic 11S globulins from tree nuts (almond, hazelnut, pistachio and walnut) but not from peanut. All the patients included in the study had positive skin prick test to tree nuts and/or peanut and we subdivided them into two different groups according to their clinical symptoms after ingestion of such allergenic sources. We showed that 11S globulins contain conserved IgE epitopes involved in cross-reactivity among mustard, tree nuts and peanut as well as species-specific IgE epitopes.

**Conclusions:**

The allergenic 11S globulin Sin a 2 from mustard is involved in cross-reactivity at the IgE level with tree nuts and peanut. Although the clinical relevance of the cross-reactive IgE epitopes present in 11S globulins needs to be investigated in further detail, our results contribute to improve the diagnosis and management of mustard allergic patients sensitized to Sin a 2.

## Background

IgE-mediated food allergy is an important worldwide health problem of increasing prevalence affecting up to 2-10% of the population [1,2]. More than 170 foods have been reported to induce allergic responses being peanuts, tree nuts, eggs, milk, fish, shellfish, wheat and soy responsible for the vast majority of reactions. Mustard is one of the most frequent spices causing IgE-mediated food allergy, and together with celery, sesame, lupine and shellfish is considered among the most significant allergenic sources in European countries [3]. The ingestion of mustard seed flour or manufactured foods containing this spice has been frequently associated with the development of severe symptoms such as generalized urticaria, angioedema or anaphylaxis in hypersensitive patients [4-7]. Mustard is worldwide consumed in home-made meals and added as a hidden condiment in many sauces, salad dressings or manufactured and processed products, which makes difficult to avoid the ingestion of this spice and increases the risk of suffering unexpected life-threatening reactions [8]. Therefore, mustard content must be declared according to the European Union directive for food labelling [9]. Yellow mustard (*Sinapis alba* L.) is commonly used in Europe whereas oriental mustard (*Brassica juncea*) is the spice used in United States and Asia.

Four allergens from yellow mustard seeds have been identified, purified and characterized so far: i) 2S albumin Sin a 1 (14 kDa) [10,11]; ii) 11S globulin Sin a 2 (51 kDa) [12,13]; iii) LTP Sin a 3 (12 kDa) [14]; and iv) profilin Sin a 4 (13–14 kDa) [14]. Sin a 1 and Sin a 3 but not Sin a 4 might act as genuine food allergens able to reach the gut immune system due to their high structural and immunological stability [15]. The capacity of Sin a 2 to act as primary sensitizer at the intestinal mucosa has not been investigated so far. We also demonstrated that Sin a 1 is a diagnostic marker for sensitization to mustard, Sin a 2 is a marker to predict severity of symptoms, and Sin a 3 and Sin a 4 are allergens associated with sensitization to other plant-derived foods from the Rosaceae family and pollens [16]. It has been reported that more than 50% of patients allergic to mustard present hypersensitivity to other different vegetable foods, mainly nuts and legumes, but whether this is due to cross reactivity and the allergens involved in such processes is an aspect that needs further investigations [16-18]. At this regard, 11S globulins might represent good candidates. Allergenic members of this protein family have been characterized from almond (Pru du 6) [19], hazelnut (Cor a 9) [20,21], peanut (Ara h 3) [22], walnut (Jug r 4) [23], pistachio (Pis v 2) [24], soybean (glycinins G1-G2) [25], Brazil nut (Ber e 2) [26], cashew nut (Ana o 2) [27], sesame seeds (Ses i 6) [28] or pecan (Car i 4) [29]. Some studies have previously shown that 11S globulins are involved in cross-reactivity between coconut and walnut [30], among buckwheat, poppy and hazelnut [31] or between peanut and other different seeds spices [32]. In addition, two more detailed studies at the molecular level based on the three-dimensional (3D) structure of the allergens and identification of IgE-binding sites suggested that Jug r 4 shows cross-reactivity with Car i 4 [29] or with Cor a 9 and other 11S globulins [33,34]. Despite these reported data, there is still little evidence of IgE-cross reactivity involving 11S globulins, a family of proteins with an overall sequence identity under 40%.

In the present study, we sought to investigate the potential implication of the 11S globulin Sin a 2 in cross-reactivity involving mustard, tree nuts and peanut. Our results show that the allergenic 11S globulin Sin a 2 shares IgG epitopes with homologous counterparts from almond, hazelnut, pistachio and walnut but not from peanut and that Sin a 2 is involved in IgE cross-reactivity with tree nuts and peanut.

## Methods

### Patients allergic to mustard

The patients included in this study were a subgroup of well-characterized patients allergic to mustard [16] who presented specific IgE antibodies against purified 11S globulin Sin a 2 as determined by both enzyme-linked immunosorbent assay (ELISA) and skin prick test (SPT). Allergy to mustard was diagnosed as previously described [35]. During the patient consultation a questionnaire gathering clinical information was filled out by an allergist. Clinical features of the patients are shown in Table 1. Although not all of the patients were allergic to tree nuts and/or peanut, all of them had positive SPT to at least one of these allergenic sources. We pooled patient’s sera into two different groups: i) Group 1, patients without clinical symptoms to tree nuts and/or peanut; ii) Group 2, patients with clinical symptoms to some of these allergenic sources (Table 1, Table 2). Serum samples from all the patients were collected and storage at −20°C until used. Sera from a non-atopic subject and from an olive pollen-allergic patient were used as controls. The study was approved by the Fundación Jiménez Díaz Ethic Committee (Madrid), and written informed consent was obtained from all subjects.

**Table 1:** Clinical characteristics of patients with mustard allergy sensitized to Sin a 2

**Patient Nº**		**Sex/age (y)**	**Mustard Symptoms**	SPT*		**Other food allergies**	SPT*					**Tree nuts/ peanut Symptoms**
				**Mustard**	**Sin a 2**		**Almond**	**Hazelnut**	**Pistachio**	**Walnut**	**Peanut**	
GROUP 1	1	F/51	OAS, U, AE, D	221	69	no	16	19	Neg	9	Neg	-
	2	M/50	OAS, AE, D	97	52	no	Neg	Neg	Neg	15	Neg	-
	3	M/29	OAS, U, RC, D	87	37	r, l, m	Neg	30	22	36	29	-
	4	M/36	OAS, U	137	139	r, m	20	10	20	18	17	-
	5	F/54	OAS, U, TT, A, RC, D	101	35	r, k	Neg	Neg	Neg	18	39	-
GROUP 2	6	F/30	OAS,CU,TT, D	119	46	k, n	15	Neg	Neg	9	7	OAS
	7	M/30	OAS,U, AE, TT, A, RC, D	103	50	r, n, p	Neg	Neg	14	Neg	Neg	OAS
	8	M/30	OAS, U, AE, TT, A, RC	171	83	n	21	14	11	8	17	OAS, RC, A
	9	M/30	OAS, AE, A, RC	169	22	r, k, n, p, m	21	82	35	124	38	OAS, AE, TT
	10	F/31	OAS, AE, CU, TT, A	55	25	r, k, n, p, m	23	57	40	59	64	OAS, AE, TT, RC
	11	M/6	OAS, TT (local)	73	8	k, n	Neg	56	Neg	54	Neg	AE, U

*M/F*, male/female; *A*, asthma; *AE*, angioedema; *CU*, contact urticaria; *D*, digestive symptoms; *OAS*, oral allergy syndrome; *RC*, rhinoconjuctivitis; *U*, urticaria; *TT*, throat tightness; *k*, kiwi; *l*, legumes; *m*, melon; *n*, nuts (including almond); *p*, peanut; *r,* r*osaceae* family (excluding almond); Neg, negative (< 0.100 for ELISA and wheal area < 7 mm^2^ for SPT).

* Skin prick test: wheal area in mm^2^.

Group 1: Tolerant to tree nuts and peanut.

Group 2: Symptoms with tree nuts and/or peanut.

**Table 2:** Specific IgE to mustard extract and purified mustard allergens by ELISA

		**ELISA‡ OD**_**492**_				
**Patient Nº**		**Mustard**	**Sin a 1**	**Sin a 2**	**Sin a 3**	**Sin a 4**
GROUP 1	1	3.366	3.186	0.980	Neg	Neg
	2	1.041	0.590	0.787	Neg	Neg
	3	2.809	2.648	0.466	1.580	0.857
	4	3.500	3.500	2.138	Neg	Neg
	5	0.847	0.553	0.529	Neg	Neg
GROUP 2	6	3.477	3.532	2.138	Neg	Neg
	7	1.327	1.153	0.119	Neg	Neg
	8	0.934	0.920	0.166	Neg	Neg
	9	0.856	0.643	0.162	0.117	Neg
	10	0.441	0.398	0.111	1.965	0.979
	11	1.508	0.738	0.216	Neg	Neg

‡ Specific IgE determined in ELISA as OD at 492 nm. Neg, negative (< 0.100).

Group 1: Tolerant to tree nuts and peanut.

Group 2: Symptoms with tree nuts and/or peanut.

### Yellow mustard seeds extract, purified Sin a 2 and rabbit anti-Sin a 2 serum

Yellow mustard seeds, almond, hazelnut, pistachio, walnut and roasted peanut protein extracts were obtained as previously described for yellow mustard seeds [12]. The allergenic 11S globulin Sin a 2 was purified from yellow mustard seeds extract as described [12].

The specific anti-Sin a 2 serum was prepared by immunizing a New Zealand white rabbit with purified Sin a 2 by weekly injection of the protein in complete Freund’s adjuvant. After 21 days of treatment the serum was obtained by centrifugation of the blood.

### Skin-prick tests

SPT were performed in all patients according to standard procedures [36]. The panel of commercial food extracts included peach, chestnut, soy, sunflower seed, almond, hazelnut, peanut, walnut, pine nut, pistachio, chickpea, lentil and bean. SPT with kiwi and apple were performed by the prick-prick method as described [37]. SPT with home-made mustard (*Sinapis alba* L.) extract (50 μg/ml) and with purified natural Sin a 2 (10 μg/ml) were performed. Histamine dihydrochloride (10 mg/ml) and physiologic saline solutions were used as the positive and negative controls, respectively. A wheal area <7 mm^2^ was considered as negative. We included almond as a nut (instead of a Rosaceae fruit), and considered peanut separately.

### Electrophoresis and immunoblotting

SDS-PAGE was performed in 15% polyacrylamide gels. Proteins (0.5 μg/lane of purified proteins or 50 μg/lane of protein extracts) were visualized by Coomassie Blue or alternatively transferred to nitrocellulose membranes (Amersham, Buckinghamshire, United Kingdom). The protein concentration was determined using the method of bicinchoninic acid (Pierce Chemical Co, Rockford, Ill, USA).

Immunodetection of proteins in membranes was achieved as described [14] by using different pool of sera from patients allergic to mustard, (diluted 1/5), or rabbit specific anti-Sin a 2 serum (diluted 1/100000). The binding of human IgE was detected by mouse anti-human IgE antibodies, provided by ALK-Abelló (Madrid, Spain), diluted 1/5000, followed by horseradish peroxidase-labelled goat anti-mouse IgG (diluted 1/5000; Pierce). Reaction to anti-Sin a 2 serum was detected by horseradish peroxidase-labelled goat anti-rabbit IgG (diluted 1/3000; BioRad, Richmond, CA). The signal was developed by using the ECL-Western blotting reagent (Amersham). For the IgG and IgE-inhibition experiments in immunoblotting, the pools of sera (diluted 1/5) or the rabbit anti-Sin a 2 serum (diluted 1/100000) were preadsorbed with 1 mg/ml of yellow mustard seeds, almond, hazelnut, walnut, pistachio or peanut extracts or with 20 μg/ml of purified Sin a 2 for 2h prior to membrane incubation as described [38]. Bovine serum albumin (BSA) was used as negative controls of inhibition. Volummograms of the reactive bands were analysed by scanning densitometry using the computer program Multigauge V3.0.

### ELISA experiments

IgG quantitation was performed by ELISA in microtiter plates (Costar, Corning, NY, USA) coated with 100 μl/well of purified Sin a 2 (2 μg/ml) or yellow mustard seeds extract (20 μg/ml) [14]. Plates were incubated with increasing dilutions of the rabbit anti-Sin a 2 serum for titration. Then, the plates were incubated with horseradish peroxidase-labelled goat anti-mouse IgG as describe above and peroxidase reaction was developed using fresh enzyme substrate and measuring optical density (OD) at 492 nm. Each value was calculated as the mean of 2 determinations after blank subtraction.

For IgG-inhibitions ELISA, after being coated with 100 μl of Sin a 2 (2 μg/ml) or yellow mustard seeds extract (20 μg/ml), the plates were incubated with the rabbit anti-Sin a 2 serum (diluted 1/100000) previously preadsorbed with increasing amounts of Sin a 2 and mustard extracts as inhibitors for 2 h [38]. Then, the plates were incubated with horseradish peroxidase-labelled goat anti-mouse IgG and peroxidase reaction developed as described above. The percentage of inhibition was determined according to the formula: % Inhibition = (1- (OD_492nm_ with inhibitor/OD_492nm_ without inhibitor)) x 100. All the determinations were carried out as duplicates.

### Sequence alignment and three-dimensional modelling

Multiple sequence alignments of Sin a 2, Pru du 6, Cor a 9, Pis v 2, Jug r 4 and Ara h 3 (accession numbers Q2TLW0, E3SH28, Q8W1C2, B7P073, Q2TPW5 and O82580, respectively) were performed with the Clustal Omega program [39]. Amino acid sequences of all 11S globulins are shown without signal peptide. Location of the signal peptide cleavage sites were predicted using SignalP 4.0 Server [40]. The 3D structure of Sin a 2 was modelled by using the services of the Swiss-Model Protein Modelling Server [41] and the structure of the soybean legumin Gly m 6 (PDB code 1od5A) [42] as template. The 3D structure of Ara h 3 correspond to PDB: 3c3v. Graphical processing of the three-dimensional structures was accomplished with PDB viewer PyMOL program.

## Results and discussion

### Clinical features of mustard-allergic patients sensitized to Sin a 2 suggest that 11S globulins might be involved in cross-reactivity with tree nuts and/or peanut

By linking the clinical features of 34 well-characterized patients allergic to mustard with component-resolved approaches we previously showed that 2S albumin Sin a 1 is a marker of genuine sensitization to mustard, 11S globulin Sin a 2 is associated to severe symptoms after mustard ingestion and LTP Sin a 3 and profilin Sin a 4, two well-known panallergens, are associated with sensitization to other plant-derived foods from the Rosaceae family and pollens [16]. Although more than 50% of mustard allergic patients are also sensitized to tree nuts and/or peanut, significant clinical associations between sensitization to Sin a 2 and being allergic to other tree nuts or peanut were not found within our cohort of mustard allergic patients [16]. However, considering that recent studies suggested that 11S globulins are implicated in cross-reactivity among taxonomically unrelated tree nuts and peanut [30-33] and that different members of this protein family have been reported as relevant allergens [19-24], we investigated at the IgG and IgE level whether the 11S globulin Sin a 2 might be implicated in cross-reactivity among mustard, tree nuts and peanut within mustard allergic patients specifically sensitized to Sin a 2. We included in the study the 11 patients allergic to mustard with the general clinical characteristics summarized in Table 1. All patients presented allergic reactions within the first 30 minutes after mustard consumption and the inclusion criteria was based on positive specific IgE to yellow mustard seed extract and Sin a 2 as determined by SPT and ELISA (Tables 1 and 2). In line with our previous data, 10 of the 11 patients sensitized to Sin a 2 developed immediate systemic reactions after the ingestion of mustard and 7 attended the emergency room, where epinephrine was administrated [16]. All the patients included in the study had also positive SPT to tree nuts (almond or hazelnut or pistachio or walnut) or peanut and 6 of them (# 6, 7, 8, 9, 10 and 11) suffered from symptoms after ingestion of tree nuts (3 of them also with peanut, # 2, 4 and 5). Interestingly, only 3 patients showed positive specific IgE to the previously known cross-reactive mustard allergens Sin a 3 and 2 to Sin a 4 (Table 2), suggesting a potential role of Sin a 2, at least in the observed positive SPT to tree nuts and/or peanut. To further analyse the implication of Sin a 2 in cross-reactivity at the IgG and IgE level and to determine potential clinical relevance within the mustard allergic patients sensitized to Sin a 2, we obtained a rabbit anti-Sin a 2 serum and pooled patients’ sera into two different groups: i) Group 1, patients with positive SPT to tree nuts and/or peanut extracts without clinical symptoms to these allergenic sources; ii) Group 2, patients with positive SPT to tree nuts and/or peanut extracts with clinical symptoms to some of these allergenic sources (Table 1).

### IgG epitopes of the 11S globulin Sin a 2 are present in almond, hazelnut, pistachio and walnut but not in peanut extracts

**Figure 1:**
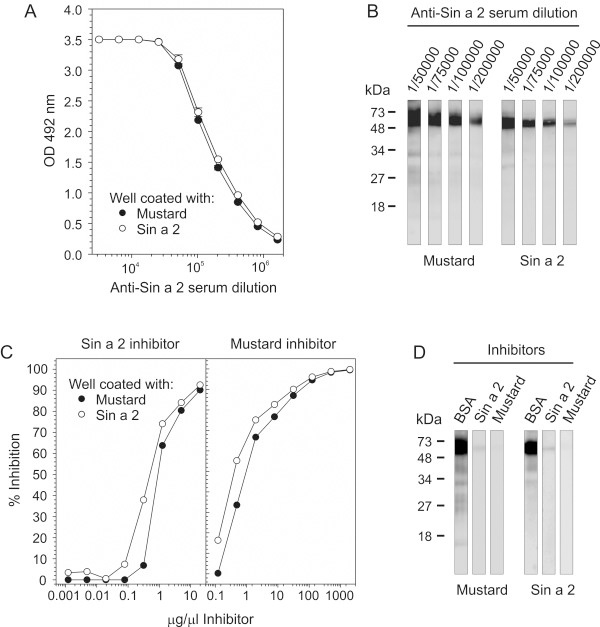
**Characterization and validation of the rabbit anti-Sin a 2 serum. A**) ELISA titration of Sin a 2 (0.2 μg/well) and mustard extract (20 μg/well) using the rabbit specific anti-Sin a 2 serum. **B**) Immunoblotting of mustard extract (50 μg/lane) or purified Sin a 2 (0.5 μg/lane) using different dilutions of the anti-Sin a 2 serum. Molecular mass markers are displayed in the left margin. **C**) ELISA-inhibition experiments of the IgG-binding to Sin a 2 or mustard extract-coated wells with increasing concentrations of Sin a 2 or mustard as inhibitors. **D**) Immunoblotting-inhibition experiments of the IgG-binding to nitrocellulose blotted mustard extract (50 μg/lane) or Sin a 2 (0.5 μg/lane) using as inhibitors BSA (20 μg/mL), Sin a 2 (20 μg/ml) or mustard (1 mg/ml). Molecular mass markers are displayed in the left margin.

**Figure 2:**
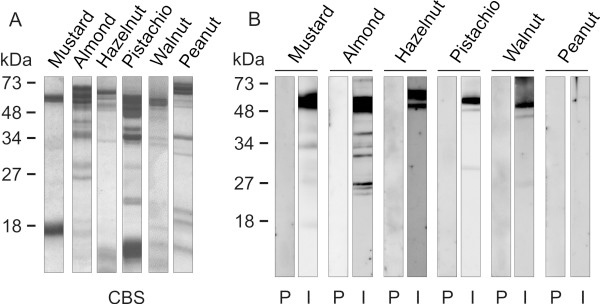
**Identification of IgG cross-reactive 11S globulins homologous to Sin a 2 in tree nut extracts. A**) SDS-PAGE and Coomassie Blue staining (CBS) of 40 μg/lane of mustard, almond, hazelnut, walnut, pistachio and peanut protein extracts. **B**) Reactivity of the anti-Sin a 2 serum diluted 1/50000 (lanes I) to mustard, almond, hazelnut, walnut, pistachio and peanut protein extracts (50 μg/lane). A pre-immune rabbit serum (lanes P) was used at the same dilution as negative control. Molecular mass markers are displayed in the left margin.

**Figure 3:**
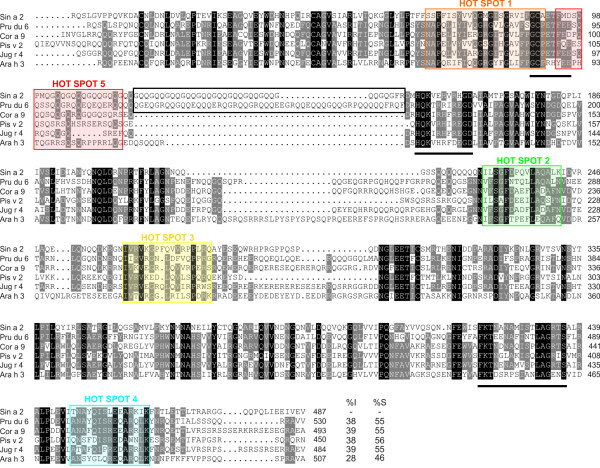
**Alignment of sequences of allergenic 11S globulins.** Yellow mustard seeds (Sin a 2), almond (Pru du 6), hazelnut (Cor a 9), pistachio (Pis v 2), walnut (Jug r 4) and peanut (Ara h 3) (UniProt accession numbers Q2TLW0, E3SH28, Q8W1C2, B7P073, Q2TPW5 and O82580, respectively). (−) for gaps. Residues conserved in all proteins are in black, and those conserved in at least 4 sequences are in grey. Underlined regions correspond to partially conserved amino acid sequences in the compared 11S globulins with the exception of Ara h 3. The sequences contained in the white square represent the additional segment of sequence enriched in Gly and Gln only presented in Sin a 2 and Pru du 6. Colored squares contain the five sequence regions previously described as IgE hot spots in 11S globulins. %I and %S, percentages of identity and similarity, respectively. Amino acid numbering for each sequence is given in the right margin.

**Figure 4:**
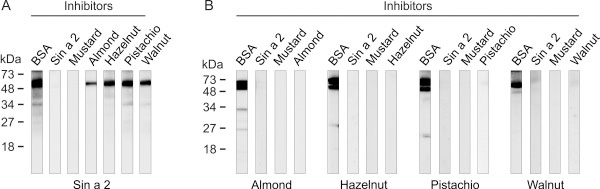
**IgG-inhibition experiments in immunoblotting.** Inhibition experiments of the IgG-binding of the rabbit anti-Sin a 2 serum (1/100000 diluted) to **A**) Sin a 2 and **B**) almond, hazelnut, pistachio and walnut protein extracts (50 μg of total protein per lane). BSA (20 μg/ml), Sin a 2 (20 μg/ml), mustard, hazelnut, pistachio or walnut protein extracts (1 mg/ml) were used as inhibitors. Molecular mass markers are displayed in the left margin.

Compelling experimental evidence demonstrated that the use of well-defined purified allergens is very useful to improve diagnosis and treatment of allergic diseases [43,44]. The availability of purified allergens allows the obtaining of specific polyclonal antibodies in mice or rabbit, which are key tools in the identification of potential cross-reactive homologous allergens in taxonomically related and non-related allergenic sources [14,45]. We employed purified Sin a 2 to raise a rabbit anti-Sin a 2 serum as described in the methods section. The specific anti-Sin a 2 serum was titrated against purified Sin a 2 and yellow mustard seeds extract by ELISA and immunoblotting (Figure 1A and B). As shown in Figure 1A, the rabbit anti-Sin a 2 serum displayed equal and very high affinity to both purified Sin a 2 and mustard extract, demonstrating that purified Sin a 2 conserved the IgG epitopes and that the employed rabbit anti-Sin a 2 serum is specific to this allergen. The anti-Sin a 2 serum also recognized the protein band at around 51 kDa, corresponding to Sin a 2, in the mustard extract and the purified allergen in a dose-dependent manner in immunoblotting (Figure 1B). When the rabbit anti-Sin a 2 serum was employed at the highest concentration (dilution 1/50000), protein bands at around 30–32 kDa were slightly detected in both cases. These protein bands, which are also recognized by sera from mustard allergic patients, were previously identified as proteolytic fragments of Sin a 2 in mustard extract [13]. To further verify the specificity of the anti-Sin a 2 serum we performed IgG-inhibition experiments in ELISA and immunoblotting (Figure 1C and D). As shown in these figures, both purified Sin a 2 and mustard extract were able to block IgG binding to whole mustard extract or to Sin a 2-coated wells in a similar manner (Figure 1C). Complete inhibition of the binding of the anti-Sin a 2 serum to mustard extract or to purified Sin a 2 was reached when purified Sin a 2 or whole mustard extract were employed as inhibitors (Figure 1D). Collectively, all these data showed that the raised rabbit anti-Sin a 2 serum displays high affinity and specificity for the allergenic 11S globulin Sin a 2. Next, we tested the presence of IgG epitopes common to Sin a 2 in tree nut (almond, hazelnut, pistachio and walnut) and peanut extracts (Figure 2). These tree nuts and peanut extracts were selected because they were the most relevant allergenic sources to which the patients included in the study were allergic to or had positive SPT (Table 1). In Figure 2A, the Coomassie Blue staining after SDS-PAGE is displayed to visualize the protein content of the different extracts. The anti-Sin a 2 serum (lanes I) but not the pre-immune one (lanes P) reacted with protein bands at around 48–52 kDa in all the tested tree nut extracts. No reaction was detected when peanut extract was assayed. These results demonstrated that the previously described allergenic 11S globulins from almond (Pru du 6),[19] hazelnut (Cor a 9) [20], pistachio (Pis v 2) [24] and walnut (Jug r 4) [23] but not from peanut (Ara h 3) [22] share common IgG epitopes with Sin a 2. As shown in Figure 3, despite showing quite low percentages of identity (ranging between 28 and 39%) and similarity (between 46 and 56%), the compared 11S globulins present regions with conserved amino acid sequences such as for example those encompassing Gly^89^-Asp^96^, His^155^-Asp^165^ or Phe^420^-Ser^436^ (underlined in Figure 3). Ara h 3 is the 11S globulin showing the lowest identity (28%) and similarity (46%) with Sin a 2 and significant differences in the amino acid sequences of the above mentioned regions are also observed (Figure 3). Therefore, the presence of IgG binding sites at these regions, which do not completely overlap with the previously identified IgE hot spots (HS), might well justify the lack of reactivity of the anti-Sin a 2 serum against Ara h 3. To complement these data, we performed IgG-inhibition experiments in immunoblotting using BSA, purified Sin a 2 or mustard as controls, and almond, hazelnut, pistachio or walnut as potential inhibitors of the IgG binding to purified Sin a 2 (Figure 4A). The reactivity to Sin a 2 of the anti-Sin a 2 serum preadsorbed with purified Sin a 2 or mustard extract was nearly abolished (95 and 99% of inhibition respectively, quantified by scanning densitometry). When tree nut extracts were employed as inhibitors, an important reduction of the IgG-binding to Sin a 2 was also reached in all the cases. Almond and walnut were the extracts showing the highest inhibitory capacity (77% and 60% of inhibition, respectively) followed by hazelnut (53%) and pistachio (43%) as determined by scanning densitometry. Sin a 2 displays a region enriched in Gln and Gly (positions Gly^118^-Arg^152^, Figure 3), which is contained exclusively in Pru du 6. If this segment bears IgG binding sites, it might justify the highest percentage of inhibition reached by almond extract. As expected considering that the specific rabbit serum was raised against purified Sin a 2, the IgG binding to almond, hazelnut, pistachio and walnut was completely abolished when purified Sin a 2, mustard or the corresponding tree nut extract were used as inhibitors (Figure 4B). Collectively, all these results firmly demonstrated that Sin a 2 shows cross-reactivity with the allergenic 11S globulins from almond, hazelnut, pistachio and walnut at the IgG level, indicating that despite their low percentage of sequence identity they share IgG epitopes likely located in the most conserved regions of the protein family.

### IgE cross-reactivity among allergenic 11S globulins from mustard, tree nuts and peanut

**Figure 5:**
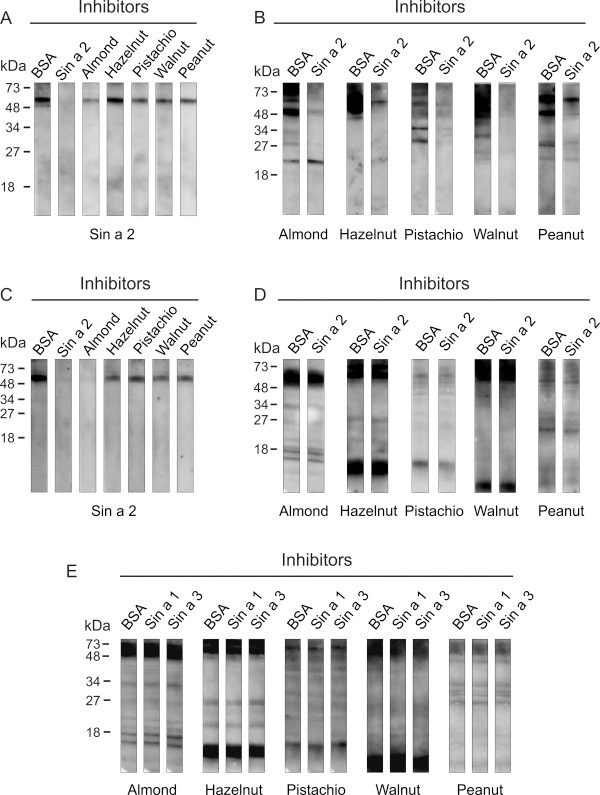
**IgE-inhibition experiments in immunobloting. A**) Inhibition of the IgE-binding to purified Sin a 2 of the pool of sera (1/5 diluted) from the group 1 of patients using as inhibitors BSA (20 μg/ml), Sin a 2 (20 μg/ml), almond, hazelnut, pistachio, walnut and peanut protein extracts (1 mg/ml). **B**) Inhibition of IgE-binding to almond, hazelnut, pistachio, walnut and peanut extracts (50 μg of total protein per lane) of the pool of sera from group 1 (1/5 diluted) using as inhibitors BSA (control) or purified Sin a 2 (20 μg/ml). **C**, **D** and **E**) The same type of IgE-inhibition experiments using the pool of sera from the group 2 of patients and the indicated inhibitors. Molecular mass markers are displayed in the left margin.

**Figure 6:**
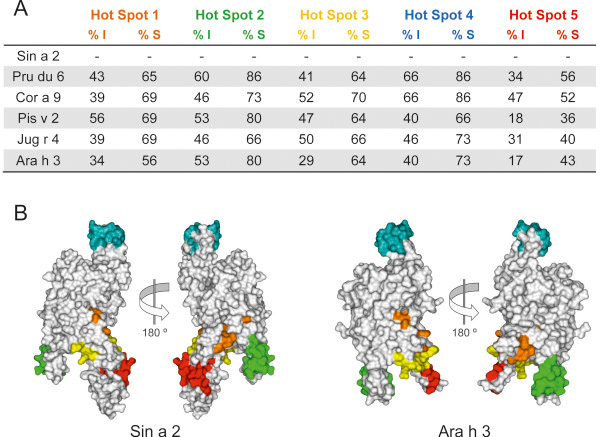
**Comparison of the IgE-binding hot spots among allergenic 11S globulins and localization on the 3D structure of Sin a 2 and Ara h 3. A**) Table showing the percentage of identity (% I) and similarity (% S) of the five IgE hot spots described in 11S globulins from almond (Pru du 6), hazelnut (Cor a 9), pistachio (Pis v 2), walnut (Jug r 4) and peanut (Ara h 3) with respect to mustard (Sin a 2). **B**) Location of the IgE hot spots on the molecular surface of the modelled 3D structure of Sin a 2 and on the 3D structure of Ara h 3. Hot spot regions on the 3D structures are colored in orange (hot spot 1), green (hot spot 2), yellow (hot spot 3), blue (hot spot 4) or red (hot spot 5).

To gain insight into the potential role of Sin a 2 in cross-reactivity involving mustard, tree nuts and peanut at the IgE level, we performed IgE-inhibition experiments in immunoblotting using purified Sin a 2 and allergenic protein extracts from mustard, different tree nuts (almond, hazelnut, pistachio and walnut) and peanut. We pooled the patients’ sera into two different groups. In the group 1 we included the 5 patients without clinical symptoms to tree nuts and/or peanut at the moment of the study and in the group 2 the 6 patients that were allergic to tree nuts (3 of them also to peanut) (Table 1). As shown in Figure 5A, the reactivity to purified Sin a 2 of the pool of sera from group 1 preadsorbed with almond, hazelnut, walnut, pistachio or peanut was inhibited at different level depending on the employed extract. By scanning densitometry we quantified that almond (63% of inhibition) was the most potent inhibitor followed by walnut, pistachio, peanut and hazelnut (49%, 34%, 32%, and 15%, respectively). Interestingly, the pool of sera from group 1 recognized allergenic 11S globulins homologous to Sin a 2 in almond, hazelnut, pistachio, walnut and peanut. The IgE-binding to these proteins was nearly totally abolished when the pool of sera was preadsorbed with purified Sin a 2 except for hazelnut and peanut (Figure 5B), which might be due to co-sensitization to species-specific epitopes from 11S globulins contained in these allergenic extracts, the existence of different allergenic isoforms or the presence of additional allergens with the same molecular weight in hazelnut and peanut. These results show that for these patients Sin a 2 is the sensitizing allergen that shares common IgE epitopes with allergenic 11S globulins from the assayed extracts, which could explain why these patients despite of not suffering from clinical symptoms to tree nuts or peanut show *in vivo* positive SPT to such allergenic sources. To further investigate the potential clinical relevance of 11S globulin Sin a 2 in cross-reactivity, we performed the same type of experiments using the pool of sera from the mustard allergic patients sensitized to Sin a 2 with clinical symptoms to tree nuts and/or peanut (group 2). As shown in Figure 5C, the IgE-binding to purified Sin a 2 was significantly inhibited by almond, hazelnut, pistachio, walnut and peanut (83%, 62%, 60%, 67%, 69% and 67% of inhibition, respectively, as determined by scanning densitometry). This result firmly confirms that Sin a 2 share common IgE epitopes with allergenic 11S globulins from tree nuts and peanuts. This pool of sera (group 2) also recognized IgE-reactive protein bands at around 48–52 kDa that were not inhibited by Sin a 2 except in the case of almond (20% of inhibition by scanning densitometry) (Figure 5D). These results together with the fact that the inhibition of the IgE-binding to purified Sin a 2 by tree nut and peanut extracts was considerably higher for group 2 than group 1 indicated that mustard allergic patients sensitized to Sin a 2 and allergic to tree nuts and/or peanut might well be also primarily co-sensitized to 11S globulins from such allergenic sources. In addition, the pool of sera from group 2 recognized different low-molecular weight IgE-reactive protein bands (< 18 kDa) or epitopes from digested food that are not present in patient group 1 (Figure 5D and E), which could make the difference in the clinical symptoms associated with tree nuts and/or peanut ingestion. Interestingly, these IgE-reactive bands were not inhibited by 2S albumin Sin a 1 nor LTP Sin a 3 (Figure 5E), indicating that these families of allergenic proteins are not involved in the observed cross-reactivity. Collectively, our data suggest that Sin a 2 and the homologous allergenic 11S globulins from the studied tree nuts and peanut might contain both conserved cross-reactive and species-specific IgE epitopes. At this regard, several studies have previously shown the existence of five regions containing IgE-binding HS on 11S globulins from tree nuts (almond, hazelnut, cashew or walnut), peanut and soybean [19,33]. Figures 3 and 6 show the amino acid sequence alignment of such regions comparing Sin a 2, Pru du 6, Cor a 9, Pis v 2, Jug r 4 and Ara h 3 as well as the location of the five IgE-binding HS on the 3D modelling of Sin a 2 and on the 3D structure of Ara h 3 . As shown in Figure 6A, the previously proposed IgE-binding HS-2 and HS-4 are quite conserved among the compared allergenic 11S globulins with percentages of identity with respect to Sin a 2 ranging from 40% (Pis v 2 and Ara h 3 in HS-4) to 66% (Pru du 6 and Cor a 9 in HS-4). The percentage of similarity with respect to Sin a 2 was even significantly higher and ranged from 66% (Jug r 4 in HS-2 and Pis v 2 in HS-4) to 86% (Pru du 6 in HS-2 and Pru du 6 and Cor a 9 in HS-4). As visualized in the 3D modelling of Sin a 2 and in the 3D structure of Ara h 3, both HS-2 and HS-4 are located at solvent-exposed areas of the monomer, thus representing ideal potential IgE cross-reactive epitopes in allergenic 11S globulins. When comparing HS-1, HS-3 and HS-5 lower percentages of identity were observed, being the HS-5 the most dissimilar. In addition, HS-1, HS-3 and HS-5 were not completely solvent-exposed on the 3D modelling of Sin a 2 or on the 3D structure of Ara h 3 suggesting that they might well constitute potential species-specific IgE epitopes. The identification of clinically relevant IgE binding sites to which patients are sensitized will contribute to improve diagnosis and accurate endotyping, which might well subsequently lead to develop more efficient and safer patient-tailored immunotherapy approaches [46-48]. Altogether, our study indicates that mustard allergic patients sensitized to Sin a 2 could present positive SPT to tree nuts and/or peanut without clinical manifestations against these allergenic sources due to IgE cross-reactivity involving 11S globulins. This finding is especially significant as it could help to avoid wrong diagnosis due to IgE cross-reactivity without clinical relevance. The fact that we also demonstrated IgE cross-reactivity involving Sin a 2 in mustard allergic patients sensitized to this allergen with clinical symptoms to tree nuts and/or peanut suggests the possibility that patients sensitized to Sin a 2 might eventually develop clinical episodes against these allergenic sources. Although the clinical relevance of the cross-reactive IgE-binding sites of the 11S globulin Sin a 2 needs to be studied in further detail, our results contribute to improve the diagnosis and management of the patients allergic to mustard sensitized to this allergen.

## Conclusions

In this study we demonstrated at the molecular level that the 11S globulin Sin a 2, which is associated with severe reactions in mustard allergic patients, is involved in cross-reactivity among mustard, tree nuts and peanut. Sin a 2 shares IgG epitopes with allergenic 11S globulins from tree nuts (almond, hazelnut, pistachio and walnut) but not with peanut. At the IgE level, we showed that 11S globulins contain conserved IgE epitopes involved in cross-reactivity among mustard, tree nuts and peanut as well as specie-specific IgE epitopes. Due to the severity of symptoms associated to Sin a 2, the definitive demonstration of the clinical relevance and the involvement of the IgE cross-reactive epitopes of 11S globulins in triggering symptoms is an important issue that will require in depth clinical studies in future.

## Abbreviations

ELISA: Enzyme-linked immunosorbent assay; OD: Optical density; SPT: Skin-prick test; 3D: Three dimensional modelling; HS: Hot spots.

## Competing interests

The authors declare that they do not have competing interests.

## Authors’ contributions

Conceived and designed the experiments: OP and SS. Performed the in vitro experiments: SS and MA. Clinical characterization of the patients: AV and JCH. Analysed and discussed the data: OP, SS, JCH, MV and RR. Contributed reagents/materials/analysis tools: AV, JCH, OP, RR and MV. Wrote the paper: OP. All the authors read and approved the final manuscript.
